# A utility valuation study assessing the impact of postprandial glucose control on quality of life of individuals with type 1 or type 2 diabetes

**DOI:** 10.1186/s41687-018-0045-6

**Published:** 2018-04-17

**Authors:** J. Jendle, A. Sandberg, S. Buchs, P. Swinburn, M. Hadi, L. Å. Levin

**Affiliations:** 1 Faculty of Medical Sciences, Örebro University, Örebro, Sweden; 2 Novo Nordisk A/S, Vandtårnsvej 114, DK-2860 Søborg, Denmark; 3 Patient-Centered Outcomes, Mapi, London, UK; 4 Department of Medical and Health Sciences, Linköping University, Linköping, Sweden

**Keywords:** Diabetes, Post-prandial hyperglycaemia, Utility

## Abstract

**Background:**

Consideration of health-related quality of life (HRQOL) in diabetes has been associated with long-term and short-term complications such as hypoglycaemia, but not with short-term glucose control. This study aimed to collect health utilities related to different degrees of poorly controlled postprandial glucose (PPG) and its impact on HRQOL in the UK and in Sweden.

**Methods:**

Three health state descriptions were developed based on literature reviews and interviews with people with diabetes and healthcare professionals, characterising mild, moderate and severe impact of postprandial hyperglycaemic symptoms on HRQOL. Time Trade-Off (TTO) interviews with a 10-year trade-off period were conducted with samples of the UK general public and of Swedish people with diabetes. Mean TTO-derived health state values were expressed on a scale from 0 (death) to 1 (full health).

**Results:**

One hundred fifty participants from the general population were interviewed in the UK (57% female, mean age 35 years) and 150 participants with diabetes in Sweden (64% female, mean age 51 years, 42% type 1 and 58% type 2 diabetes). The mean TTO-derived health state values were for the UK and Swedish participants: mild impact of poorly PPG control (0.89/0.76); moderate (0.75/0.71); severe (0.56/0.58).

**Conclusions:**

Glucose lowering treatments associated with improved control over PPG levels could have important benefits to people with type 1 and type 2 diabetes since findings suggest that increasing severity in postprandial hyperglycaemic symptoms is perceived as having significant negative impact on HRQOL of individuals with type 1 or type 2 diabetes.

## Background

The aim of diabetes treatment is to achieve good glycaemic control to reduce the risk of both short- and long-term complications [1]. Poor glycaemic control can lead to a higher risk of microvascular and macrovascular complications [2, 3]. Poor glycaemic control has been shown to be ultimately linked to an increased risk of mortality [4]. Control of postprandial glucose (PPG) is considered necessary to achieve recommended glycaemic goals [5]. PPG and HbA1c are strongly correlated, and reaching target HbA1c requires both a good fasting plasma glucose as well as PPG control [1, 6].

Most published studies report worse health-related quality of life (HRQOL) for people with diabetes compared to the general population, often largely due to diabetes-related long-term complications [7, 8]. Hypoglycaemia, a very common side effect of insulin therapy, has been associated with negative short-term consequences, such as various symptoms (confusion, loss of consciousness) and cognitive-motor disruptions [9, 10]. The short-term effects of elevated PPG and the impact of having inadequate PPG control on a frequent basis have been less well documented. Yet, post-prandial hyperglycaemia is often reported in patients with diabetes on active glucose lowering treatment [11], and poorly controlled PPG has been linked to an increased risk of macrovascular complications, including cardiovascular diseases [12]. Although the impact on HRQOL of diabetes and long-term complications has been extensively described [7, 8], the short-term effect of poor PPG control on patient functioning and HRQOL has not yet been documented. There is currently a gap in the published literature on how living with different degrees of poorly controlled PPG affects HRQOL of people with diabetes.

The present study aimed at developing health states describing how living with different degrees of poorly controlled PPG affects HRQOL in people with type 1 and type 2 diabetes, and to subsequently collect related health utilities in the UK and in Sweden. These utility values may be used to populate pharmacoeconomic models.

## Methods

### Health states development

Targeted literature reviews using Medline and Embase were undertaken to identify the impact of poor PPG control and the important issues for people with type 1 and type 2 diabetes, and to review patient-reported outcomes instruments measuring symptoms associated with hyperglycaemia. The concepts of interest searched for were symptoms, signs, impacts, burden of condition, and quality of life or HRQOL. The search required the title or abstract to include qualitative research (keywords: qualitative research, interviews, grounded theory, phenomenological, focus group, concept or endpoint model) and “hyperglycaemia” (or “postprandial”). This initial search led to the identification of 601 abstracts, however, none reported the concepts in scope. The strategy was therefore adjusted to search for patient-reported outcomes instruments assessing symptoms associated to hyperglycaemia, to better understand the daily postprandial experience of people with diabetes, and in particular those occurring after an acute hyperglycaemic event. The main topics which emerged were the symptoms of hyperglycaemia, including thirst, dry mouth, frequent urination, tiredness/weakness, irritability, poor concentration, headache, blurred vision, nausea and dizziness [13]. Another topic was the impact on cognitive performance due to varying levels of hyperglycaemia [13]. Acute hyperglycaemia was found to have significant adverse effects on cognitive functioning [14, 15]. Lastly, information was retrieved on the potential impact on mood, such as increased anxiety, tiredness and lethargy, and decreased happiness, which along with the cognitive effects can have a detrimental effect on daily activities [15, 16].

From December 2015 to January 2016, information interviews were conducted by phone with people with type 1 (*n* = 6) and type 2 diabetes (*n* = 10), expert clinicians (*n* = 6) and nurses (*n* = 2) in the UK. People with diabetes were recruited from two patient panels and health care professionals were recruited using specialist recruitment agencies. These interviews aimed to characterise the impact of postprandial hyperglycaemic symptoms. Respondents with diabetes reported experiencing symptoms such as feeling thirsty, blurred vision, tiredness, and the need to urinate frequently if their blood sugar levels were high, as well as dizziness and feeling hot and sweaty. Some also reported experiencing depression or limitations on their daily activities but most felt they had become accustomed to their health state. Respondents mentioned monitoring their glucose levels anywhere between twice a week to several times a day. The health care professionals reported symptoms similar to those described by the respondents with diabetes. The health care professionals described also an impact on patients’ daily activities, emotional well-being and social lives.

Based on the literature reviews and these interviews, three health states were drafted characterising the HRQOL impact of living with varying degrees of poor PPG control (Table 1).

**Table 1:** Health state descriptions characterising mild, moderate and severe impact of postprandial glucose (PPG) control on health-related quality of life (HRQOL)

Health state	Description
Mild impact of PPG control symptoms on HRQOL	● You have a chronic, long-term condition which requires you to monitor your blood sugar levels regularly at mealtimes. This typically involves pricking your finger with a needle to draw a drop of blood. You occasionally need to visit a clinic for a check-up. ● You do not usually experience any issues after eating although you can occasionally find yourself feeling slightly more tired than usual. ● You are capable of socializing normally but need to monitor what you eat and drink. ● You are able to exercise normally but you need to monitor your blood sugar levels more carefully when doing so.
Moderate impact of PPG control symptoms on HRQOL	● You have a chronic, long-term condition which requires you to monitor your blood sugar levels regularly at mealtimes. This typically involves pricking your finger with a needle to draw a drop of blood. You occasionally need to visit a clinic for a check-up. ● After eating, your increased blood sugar can make you feel thirsty and you may visit the toilet more often than usual. You can also feel tired and need to rest. Occasionally you find it hard to concentrate. ● You may find it difficult to complete household chores or other usual activities and need to take regular breaks whilst your sugar levels remain high. ● Your blood sugar levels can interfere with seeing family and friends. You need to carefully monitor what you eat and drink. You need to limit strenuous physical activities such as sports and you may not feel comfortable driving. ● You are irritable sometimes after eating. You can experience depression due to your condition and the need to monitor your blood glucose levels regularly.
Severe impact of PPG control symptoms on HRQOL	● You have a chronic, long-term condition which requires you to monitor your blood sugar levels several times in the day. This typically involves pricking your finger with a needle to draw a drop of blood. You regularly need to visit a clinic for a check-up. ● After eating, your increased blood sugar usually makes you feel thirsty and you need visit the toilet more often than usual. As a result of going to the toilet more you may not sleep well at night and feel tired the next day. You frequently feel tired and need to rest. You often find it hard to concentrate and can experience periods of blurred vision. ● You find it difficult to complete household chores or other usual activities and need to take regular breaks whilst your sugar levels remain high. You need to avoid strenuous physical activities. ● Your blood sugar levels can interfere with seeing family and friends. You need to very carefully monitor what you eat and drink. You need to avoid participating in physical exercise such as sports and you may not feel comfortable driving. ● You are often irritable after eating. You feel depressed due to your condition and the need to monitor your blood glucose levels very regularly.

Validation interviews were conducted by telephone with people with type 1 diabetes (*n* = 2) and type 2 diabetes (*n* = 6), expert clinicians (*n* = 2) and one nurse in the UK. The health states were subsequently adjusted slightly to reflect the experience of patients better (e.g. the possibility of experiencing of blurred vision was added to the “severe” health state).

In a separate study, there was an opportunity to field-test the health states in a self-reported online survey with 940 respondents with type 1 or type 2 diabetes recruited from patient panels in Italy, UK and US. Respondents were specifically asked if they had experienced situations similar to the ones described in the health states. Two thirds (66.6%) had experienced the situation described in the “mild” health state, 38.5% the “moderate” health state and 26.6% the “severe” health state [17].

Lastly, members of the UK general public (*n* = 6) took part in health state pilot testing interviews. These interviews indicated that there were no issues with the understanding of the health states and were thus suitable for use in the valuation exercise. The final health state descriptions are presented in Table 1. These descriptions underwent linguistic validation in Swedish, including forward and backward translations. This ensured the health states were conceptually equivalent to the original and comparable across languages, culturally relevant to Sweden, and easily understood by Swedish people with diabetes participating in the valuation study.

### Valuation study

Due to different health technology assessment (HTA) bodies requirements of the target population to perform the valuations, the study was performed with the UK general public and with disease-experienced people in Sweden with type 1 and type 2 diabetes [18, 19]. Participants were invited across geographical areas in the UK and in Sweden to participate in the valuation study through established panels, newspaper advertisement, and word-of-mouth. Participants were aged over 18, resident in the UK or Sweden, and had given their informed consent. Participants in the UK were enrolled on the basis of age and gender to approximate the UK general public as described by available census data. Participants in Sweden were recruited according to a quota of age, gender and type of diabetes, based on the Swedish national diabetes registry.

First, participants were asked to complete a brief socio-demographic form and the EQ-5D-3L, to document the characteristics and general health status of the participants. The EQ-5D-3L is a self-administered standardised generic instrument for measurement of health outcomes [20]. From the EQ-5D-3L a single utility index score was derived, using the UK value set. EQ-5D index score ranges from less than 0 (health state equivalent to death) to 1 (best possible health). The instrument also includes a visual analogue scale (VAS), assessing general health status and ranging from 0-worst to 100-best imaginable health state.

To familiarise the participants with the descriptions and to provide a preliminary indication of the ranking of the health states, the participants ranked them using a 100-point VAS scale running from 0 (worst) to 100 (best). Participants were also asked to rank a health state called ‘dead’, to indicate whether any state would be assessed as “worse than dead”. Each health state was presented to the participant on separate cards. All health states were identified with a symbol so that no reference was made to the name of the health state.

The time trade-off (TTO) exercise began by rating health states that were assessed as ‘better than dead’ (i.e. scored above ‘dead’ on the VAS) [21]. Participants were asked to imagine that they were in a selected health state for a 10-year period. For each health state, they could choose between two alternatives: remaining in the health state without improvement for ten years, or trading a number of the ten years off for a hypothetical treatment that would restore the person to full health. The process incorporated a ‘ping-pong’ approach with duration traded back and forth between higher and lower values that were iteratively narrowed to the point of indifference between the suggested alternatives. Depending upon where this point occurred, a utility value was assigned. Once the health states identified as ‘better than dead’ were all valued, a modified approach was undertaken with health states ‘worse than dead’. In this instance the trade-off occurred between the two alternatives being dead and spending a defined time in a particular health state followed by a period of full health. Health state utility values are expressed on a cardinal scale of 0–1 (0 indicating death; 1 indicating full health). A negative value between − 1 and 0 can be obtained for health states ranked worse than death.

### Statistical analysis

Quantitative (continuous) variables were described by their mean, standard deviation (SD), and minimum and maximum values. Qualitative (categorical) variables were described by the frequency of each response choice.

Analyses were conducted on the entire country datasets. In addition, a series of subgroup analyses were performed in Swedish datasets to describe VAS and TTO values by diabetes type and disease duration.

All data processing and analyses were performed with SAS® software for Windows Version 9.2 or later (SAS Institute, Inc., Cary, NC, USA).

## Results

### Study population

Participant characteristics are presented in Table 2. The mean age of the participants in the UK (*n* = 150) was 35 years; 57% were women, the mean EQ-5D VAS score reported was 81.5 and the mean EQ-5D index score was 0.90.

**Table 2:** Participant characteristics and EQ-5D visual analogue scale (VAS) and index scores by country and diabetes type (UK general public *n* = 150; Swedish people with diabetes *n* = 150)

Characteristics	UK general public	Swedish people with diabetes		
	Overall (*n* = 150)	Type 1 diabetes (*n* = 63)	Type 2 diabetes (*n* = 87)	Overall (*n* = 150)
Age - years, mean (SD)	34.8 (12.3)	39.2 (17.2)	60.3 (14.4)	51.5 (18.8)
Min - max	18–87	18–82	19–86	18–86
Gender - Female, *n* (%)	86 (57.3)	43 (68.3)	53 (60.9)	96 (64.0)
Qualifications, *n* (%)				
University education	93 (62.0)	13 (20.6)	22 (25.3)	35 (23.3)
Missing	2 (1.3)	0 (0.0)	0 (0.0)	0 (0.0)
Main activity, *n* (%)				
Employed full-time	96 (64.0)	32 (50.8)	19 (21.8)	51 (34.0)
Employed part-time	22 (14.7)	5 (7.9)	11 (12.6)	16 (10.7)
Student	13 (8.7)	9 (14.3)	2 (2.3)	11 (7.3)
Seeking work/unemployed	5 (3.3)	6 (9.5)	6 (6.9)	12 (8.0)
Retired	3 (2.0)	11 (17.5)	49 (56.3)	60 (40.0)
Other	10 (6.6)	0 (0.0)	0 (0.0)	0 (0.0)
Missing	1 (0.7)	0 (0.0)	0 (0.0)	0 (0.0)
Age at diagnosis - years, mean (SD)	–	20.8 (15.2)	49.7 (13.9)	37.4 (20.4)
Duration of diabetes - years, mean (SD)	–	18.3 (13.1)	10.6 (9.8)	13.9 (11.9)
EQ-5D VAS score, mean (SD)	81.5 (14.7)	76.7 (17.8)	72.1 (15.7)	74.1 (16.7)
EQ-5D Index score, mean (SD)	0.90 (0.17)	0.81 (0.28)	0.71 (0.35)	0.75 (0.32)

*SD* standard deviation

The mean age of the Swedish participants (*n* = 150) was 51 years; about two thirds of the sample (64%) were women. Sixty-three participants (42%) had type 1 diabetes and 87 (58%) had type 2 diabetes. The mean diabetes duration was longer for participants with type 1 diabetes (18.3 years) than participants with type 2 diabetes (10.6 years). Swedish participants reported a mean EQ-5D VAS score of 74.1 and a mean EQ-5D index score of 0.75. The EQ-5D VAS score and index score were slightly higher in participants with type 1 diabetes than participants with type 2 diabetes.

### Health state valuation

In the UK analysis set, for the “mild” health state, the mean VAS value was 73.9 and the mean TTO value was 0.89. For the “moderate” health state the mean VAS value was 54.5 and the mean TTO value was 0.75, and for the “severe” health state, the mean VAS value was 37.4 and the mean TTO value was 0.56 (Table 3).

**Table 3:** Time trade-off (TTO) exercises in the UK (*n* = 150) and Swedish (*n* = 150) analysis sets, presented a mean (SD)

Health state	UK general public (*n* = 150)	Swedish people with diabetes		
		Type 1 diabetes (*n* = 63)	Type 2 diabetes (*n* = 87)	Overall (*n* = 150)
Mild impact of PPG control symptoms on HRQOL				
	0.89 (0.13)	0.79 (0.22)	0.74 (0.22)	0.76 (0.22)
Moderate impact of PPG control symptoms on HRQOL				
	0.75 (0.22)	0.73 (0.24)	0.69 (0.24)	0.71 (0.24)
Severe impact of PPG control symptoms on HRQOL				
	0.56 (0.28)	0.61 (0.27)	0.56 (0.28)	0.58 (0.28)

*SD* standard deviation, *PPG* postprandial glucose, *HRQOL* health-related quality of life

In the Swedish analysis set, for the “mild” health state, the mean VAS value was 71.1 and the mean TTO value was 0.76. For the “moderate” health state the mean VAS value was 53.9 and the mean TTO value was 0.71 and for the “severe” health state, the mean VAS value was 43.0 and the mean TTO value was 0.58 (Table 3).

In both the UK and the Swedish analysis sets, the VAS and TTO values associated with the health states followed a predictable order, based upon the likely HRQOL burden associated with the descriptions of the health states. No participants in the UK or Swedish analysis sets rated any of the health states as worse than dead.

### Health state valuation by type of diabetes and duration of living with diabetes

The TTO values obtained from participants with type 1 diabetes for all health states were quite similar but consistently higher than the values obtained from participants with type 2 diabetes (Table 3). Health state valuation by duration of living with diabetes are provided for information (Table 4), however, no robust conclusions on the comparison between subgroups can be drawn due to the small sample sizes (in particular in the subgroups of participants with type 1 diabetes) and the potentially confounding impact of the age disparity between groups.

**Table 4:** Time trade-off (TTO) exercises in the Swedish (*n* = 150) analysis sets per diabetes type and duration of living with diabetes, presented as mean (SD)

Type of diabetes	Duration of living with diabetes	*N* ^a^	Mild impact of PPG control symptoms on HRQOL	Moderate impact of PPG control symptoms on HRQOL	Severe impact of PPG control symptoms on HRQOL
			TTO	TTO	TTO
Type 1	≤5 years	6	0.66 (0.28)	0.63 (0.27)	0.65 (0.27)
	6–9 years	8	0.83 (0.30)	0.66 (0.38)	0.46 (0.24)
	≥10 years	47	0.81 (0.20)	0.76 (0.22)	0.63 (0.28)
Type 2	≤5 years	26	0.75 (0.23)	0.70 (0.25)	0.62 (0.24)
	6–9 years	16	0.67 (0.26)	0.63 (0.25)	0.47 (0.29)
	≥10 years	40	0.76 (0.19)	0.71 (0.23)	0.54 (0.28)

^a^Data about the duration of living with diabetes was missing for 7 patients who were not included in the analysis per duration

*SD* standard deviation, *PPG* postprandial glucose, *HRQOL* health-related quality of life

## Discussion

This study is the first study to derive health utilities related to the short-term HRQOL impact associated with different degrees of poor PPG control in people with diabetes. Health utility data from people with diabetes has been widely collected over the past decade, primarily addressing the impact of a range of potential comorbidities [22–24]. The direct impact on HRQOL of inadequate PPG control has not been sufficiently addressed; this study is filling an evidence gap, and its findings suggest that increasing severity in elevated PPG is perceived to have significant negative consequences for the short-term HRQOL of individuals with diabetes.

The utility values elicited for the health states in the UK and Swedish samples followed expected patterns, i.e. increased burden resulting from poorer PPG control is associated with a subsequent decline in utility. The decrement of utilities between the mild and moderate state and the moderate and severe states were all above 0.05 for both the UK and Swedish samples. This 0.05 threshold has been suggested by Feeny (2005) as the meaningful difference [25].

The “mild” health state (mean utility 0.89 in the UK sample, 0.76 in the Swedish sample) was perceived as a condition which did not actually differ much from the participants’ own reported health status (0.90 and 0.75 respectively). This might not be surprising, as the description of the “mild” health state omits most of the symptoms and issues associated with increased PPG after eating, such as feeling thirsty, an increased need to urinate, and potential for self-imposed restrictions on physical activity. The derived utility value for this health state falls within the ‘well-controlled’ diabetes range reported by a previous UK study [26]. The “moderate” health state demonstrates a reduction in HRQOL (mean utility of 0.75 in the UK sample, 0.71 in the Swedish sample), as a result of the increased symptom burden and corresponds to the reported values for ‘not well-controlled’ diabetes in the earlier study [26]. From both populations, values for the “mild” and “moderate” health states fall more or less within the range of values (0.71 to 0.94) for complication-free type 2 diabetes presented in a recent systematic review of such utilities [27]. This suggests that a poor PPG control, as long as it is associated with mild or moderate symptoms, may actually form an intrinsic part of the disease experience which is perhaps not readily reported as being of a key concern to individuals. The “severe” health state demonstrates very significant consequences for patients’ HRQOL (mean utility 0.56 in the UK sample, 0.58 in the Swedish sample). Health care professionals involved in the development process described this health state as a condition indicative of an individual with substantial problems with managing and controlling the disease. Experiencing a “severe” health state appears to indicate a HRQOL burden similar to that of developing a complication as a direct result of the condition. This suggests that individuals living with a condition which is reflected by the “severe” health state have substantially impaired HRQOL, and that could represent a significant unmet need for this particular patient group.

Overall, the results obtained directly from people with diabetes in Sweden mirror those of the UK societal data collection efforts in that decreasing level of PPG control is associated with a subsequent decline in HRQOL. Two observations should still be highlighted. First, the utility values obtained from people with type 1 diabetes were consistently higher compared to the values obtained from people with type 2 diabetes. This may be explained by the duration of living with the disease being much longer for people with type 1 diabetes than for people with type 2 diabetes; people with type 1 diabetes may be more used to experiencing issues with their PPG control, this could be due to more stringent glycaemic goals and better equipped with glucometers and/or continuous glucose monitoring systems to detect a postprandial excursion compared to people with type 2 diabetes. Also since glucose fluctuations are more common in people with type 1 diabetes they may have learned to better cope with them. Second, as mentioned above, the slight difference observed for the “mild” health state between the Swedish and the UK participants, indicating that the Swedish participants perceived the burden in this health state as more severe than the UK participants, may possibly be due to patients interpreting disease-related events as a sign of poor disease control and a potentially poor prognosis.

While the recommendations by HTA bodies vary in terms of whose preference assessment should be assessed (experienced patients versus the general public), the equivalence between values from different respondents has been challenged and questioned for years. Our data should be viewed in light of a meta-analysis by Peeters et al. in 2010, which showed that patients generally provide higher valuations than members of the general public [28]. In our study we observe a different pattern; however, one should keep in mind that local context may hinder the comparability of the values across countries, and one would need to perform the valuation exercise with both groups in the same country to draw conclusions.

Normative data from a previous study of the UK population found a mean utility value of 0.86, with people below 45 years of age, on the other hand, reporting decidedly higher values (in the 0.91–0.94 range) [29]. The higher index score value in our study sample (0.90) may be partly a function of age, as indeed our study population reported a lower median age of 31 vs. 39 in the UK general population [30], and partly general improvements in health and healthcare over the last two decades since the norms were produced.

Compared to the EQ-5D index score (0.91) estimated by Burström et al. [31] in a general population sample from Stockholm (*n* = 49,169; mean age 46.2 years), there was a clear difference in self-reported HRQOL in our Swedish samples (mean EQ-5D index score 0.81 for type 1 diabetes and 0.71 for type 2 diabetes). This would be expected, given the diagnosis and the added impact of a more elderly (mean age 51.5 years) and likely more unwell type 2 diabetes population in particular. The value for type 2 diabetes is in line with the value (0.77) reported by Kiadaliri et al. for a similar patient population in Sweden [23]. Such a value for type 1 diabetes in Swedish patients was missing from their study to allow a comparison with the value from our study. We cannot exclude that the age of the participants could be a confounding factor partially contributing to the difference between the values observed for type 1 diabetes and for type 2 diabetes. Based on the 2016 yearly report of the Swedish National Diabetes Register (NDR), our sample was slightly younger than the overall diabetic population in Sweden [32]. Mean ages of individuals with type 1 and type 2 diabetes was 46.4 and 68.3 years against 39.2 and 60.3 years in our study samples.

The health utility values obtained in this study shall be used to feed economic modelling while keeping in mind that we were researching a level of control rather than the occurrence of acute events – so the vignettes describe states of being in a certain level of control, which included a different number and severity of symptoms. This study did not aim at describing the utility decrement of a single symptomatic PPG peak. The health states attempt to describe broadly certain characteristics of PPG experience and may not capture all the salient features of that experience. Even though steps were taken in the development process to try and identify those that were most common and/or most burdensome, this is unlikely to be exhaustive and the study findings should therefore be interpreted with appropriate caution. Furthermore, the aim was not to differentiate symptoms of diabetes from symptoms of PPG, but to focus on PPG symptoms and their impact as reported by the patients. Although health states were developed based on interviews with respondents only in the UK, they were confirmed in a self-reported online survey, conducted with almost a thousand respondents with diabetes in Italy, US and UK [17]. Based on this, it is assumed that the descriptions are applicable to people with diabetes in developed countries.

Second, a TTO approach with a standard 10-year time frame was used to describe how living with different degrees of poor controlled PPG affects HRQOL. It has been shown that the respondents' own subjective life expectancy (SLE) influence TTO responses in such way that the higher the respondents SLE, the lower the number of years traded off, both when asking the general public [33] or experienced patients [34]. As the SLE probably was longer than 10 years, this implies that the negative impact of PPG could be underestimated in the TTO responses. Suggestion for future research is to study the utility decrement of a single symptomatic PPG peak and the frequency of those events.

## Conclusions

This study indicates that increasing severity in elevated PPG is perceived to have significant negative consequences for the short-term HRQOL of individuals with diabetes. These findings open perspectives for the importance of developing treatment which could prevent elevated PPG and the possibilities of continuous glucose monitoring to reduce glycaemic excursions and thus may have important additional benefit to people with diabetes and PPG issues.

## Abbreviations

HRQOL: Health-Related Quality of Life
HTA: Health Technology Assessment
PPG: Postprandial Glucose
TTO: Time Trade-Off
